# Assessing the psychobiological demands of high-fidelity training in pre-hospital emergency medicine

**DOI:** 10.1186/s13049-024-01272-4

**Published:** 2024-10-09

**Authors:** Mark A. Wetherell, Glenn Williams, Jeff Doran

**Affiliations:** 1 Department of Psychology, Northumbria University, Newcastle upon Tyne, NE1 8ST UK; 2 South Tees Hospitals NHS Foundation Trust, The James Cook University Hospital, Middlesbrough, TS5 3BW UK

**Keywords:** Psychobiology, Stress, High-fidelity training, Salivary cortisol, Heart Rate, HRV-stress

## Abstract

**Background:**

Individuals who provide critical emergency care mount rapid psychobiological responses when faced with an incident. These responses are adaptive and ensure resources at time of demand; however, frequent activation with minimal opportunity for recovery can have negative consequences for health and wellbeing. Monitoring individuals in real emergency situations would provide an understanding of their stress responses during the provision of critical care; however, this presents logistical challenges. An alternative is to assess individuals during high-fidelity training scenarios. This is the first comprehensive assessment of psychobiological responding during continuous high-fidelity training in pre-hospital emergency medicine.

**Methods:**

A sample of doctors and paramedics (*N* = 27) participated during 10 days of training and a weekend of no activities. Training involved the acquisition of human factors, non-technical and surgical skills, and their application in complex high-fidelity scenarios including road-traffic accidents, firearms incidents, and swift water rescue operations. On each day participants reported levels of state, cognitive, and somatic anxiety, and self-confidence following waking and before sleep, and their anticipated (at wake) and experienced (before sleep) demands of the day. Saliva samples were obtained each day for assessment of diurnal cortisol indices and the Cortisol Awakening Response (CAR). Garmin smartwatches were worn throughout for the collection of heart rate and HRV-derived stress.

**Results:**

There were significant (*p* < 0.001) differences across days for state, cognitive, and somatic anxiety; self-confidence; anticipated and experienced demands; aggregated measures of heart rate and HRV-derived stress; levels of cortisol at waking (*p* = 0.002) and for the CAR (*p* < 0.001). Measures of psychobiological responding during training were distinct from the weekend and the highest levels of psychobiological responding occurred on days characterised by greater anticipated and experienced demands.

**Discussion:**

This high-fidelity training is typical of the day-to-day requirements of emergency services and these observations are representative of functioning during real-life critical care emergencies. Increased responding during times of demand is adaptive; however, frequent and sustained responding increases allostatic load and is a contributor to burnout. As burnout is a significant concern in emergency medicine, this study identifies patterns of responding and recovery that may impact upon longer-term health and wellbeing.

## Introduction

Situations where the perceived demands of an event exceed our perceived ability to cope are interpreted as challenging or threatening and can lead to stress [1]. To cope with these challenging events, or stressors, biological mechanisms are activated, principally the Sympathetic Adrenal Medullary (SAM) and Hypothalamic-Pituitary-Adrenal (HPA) axes, which through the secretion of the hormones adrenaline and cortisol respectively, facilitate the liberation and mobilisation of energy resources to deal with threats [2]. These biological responses are our attempts to maintain allostasis and our ability to respond is entirely adaptive. Acute activation of these responses is therefore observed immediately prior to and during challenging events as evidenced by increases in psychobiological responses to acute laboratory stressors [2] and in individuals experiencing real-life stressors, for example, skydiving [3] and firefighting [4]. In addition to acute responding, cortisol is responsible for a number of regulatory functions. These functions are maintained through a marked profile of diurnal secretion characterised by a rapid increase in the 30–45 min after awakening (Cortisol Awakening Response: CAR) followed by a decline across the day reaching nadir around midnight [5]. This profile is also associated with a range of psychosocial factors. Given the energising role of cortisol in response to acute challenges, it is suggested that the CAR has an adaptive function in preparing for forthcoming demands and provides an energetic boost to maximise functioning [6]. In support, increased CARs have been observed in situations requiring greater perceived demand. Newly qualified doctors demonstrate increased CARs at times characterised by a lack of control over the environment [7] and increased CARs have been observed on workdays compared to less stressful weekend days [8, 9]. Elevated morning cortisol has been observed on the day of challenging sporting events such as dancing, motorcycling and tennis competitions [10–12]. CARs of greater magnitudes have also been observed on the day of manipulated social and cognitively demanding stressors in ambulatory [13] and controlled sleep laboratory conditions [14].

The experience of acutely challenging events is a ubiquitous feature of those that provide critical emergency care [15]. As such, it is adaptive for individuals who provide critical emergency care to mount rapid physiological responses to provide the resources necessary to deal with the presenting situation. The nature of this occupation, however, means that these responses are likely to be frequent with limited opportunity for recovery [4], and this sustained activation could place these individuals at greater risk of psychological and physical morbidity through increased allostatic load [16]. The observation and monitoring of individuals while they are engaging in emergency situations in real time would provide an understanding of the stress responses of these individuals in a critical care scenario. Moreover, it could provide a greater understanding of the psychobiological processes that can lead to deleterious outcomes in the longer term. Such monitoring, however, presents significant logistical challenges and could compromise the delivery of care to patients. A viable alternative is to assess individuals during simulated training scenarios. Simulated training is an effective method of developing the skills required to respond appropriately in critical situations and is widely used in medical training [17]. The extent to which simulations mirror real-life situations with high fidelity is crucial for the learning experience. High-fidelity simulations typically focus on physical and conceptual fidelity, that is, the extent to which the environment and equipment are realistic representations of equivalent real-world scenarios. However, high-fidelity simulations should create realistic environments to the extent that they also elicit the emotional responses that would typically be experienced in real emergency situations [18]. In support, increases in self-reported anxiety and elevated heart rate following simulated cardiac life support training have been observed in those who experienced training with additional emotional stressors [19]. Similarly, increases in self-reported stress, sympathetic activation and cortisol secretion have been observed immediately following immersive resuscitation simulation with added emotional triggers [20]. Furthermore, those who experience additional emotional stress during training demonstrate improved recall of practical skills [21], supporting the notion that high-fidelity training that encompasses environmental, equipment, and psychological fidelity, affords a more authentic and enhanced learning experience.

Studies of simulation of medical care to date have largely assessed acute scenarios involving the treatment of single patients. For most medical practitioners mass casualty incidents involving multiple services are not frequently occurring. Consequently, there is a concomitant reduction in specific training for such events and therefore less empirical research in this area [22]. However, such incidents are more typical in emergency medicine highlighting the importance of high-quality training, and moreover, an understanding of the psychological and physiological demands and implications of such training. The ‘Pre-Hospital Emergency Medicine Crew Course’ (PHEMCC) is a world-leading training experience run by the Great North Air Ambulance Service, UK which provides this opportunity. The PHEMCC is a realistic scenario-based training course for medics. The course runs for 2 weeks, including a weekend of non-training, and provides a unique opportunity to assess the demands of high-fidelity training on psychobiological functioning. Furthermore, the nature and duration of the training allows for the assessment of scenarios that go beyond the treatment of individual patients during acute emergency situations. That is, the training involves complex, multi-patient scenarios, and the course duration allows for the assessment of the accumulative impact of delivering emergency medical care over an extended period, such as would be experienced in real world settings.

This study is the first to assess psychobiological functioning during a 2-week, high-fidelity training course in pre-hospital emergency medicine. Each training day differs in terms of its comprising activities and associated demands of learning, cognitive, and physical load, and we assessed these proposed workloads in relation to indices of psychobiological functioning. Based on existing evidence regarding stress, demand, and psychobiological responding, it is proposed that training days with different learning, cognitive and physical loads, will be characterised by differences in psychobiological responding. Specifically, we assessed differences in psychological factors associated with distress (state, somatic, and cognitive anxiety; stress; worry), and perceived resource (self-confidence; coping; control); and biological markers of the Sympathetic Adrenal Medullary (heart rate; HRV-derived Stress), and Hypothalamic-Pituitary-Adrenal (waking and before sleep cortisol levels; Cortisol Awakening Response) axes.

## Materials and methods

All materials are available to view at Open Science Framework (https://osf.io/r4cep/). This study received ethical approval from Northumbria University Ethics Board (#28196).

### Recruitment & participants

Participants were recruited from the Pre-Hospital Emergency Medicine Crew Course (PHEMCC). The PHEMCC is aimed at (1) Doctors at senior registrar or consultant level with a background in Emergency Medicine, Anaesthesia or Intensive Care and experience of delivering general anaesthesia and critical care in hospital settings and pre-existing knowledge of the management of major trauma and critically unwell patients, and (2) qualified paramedics with experience of working on the road and looking to further their critical care skills and knowledge. The course is run once per year and has a maximum cohort size of 15. Participants were given information about the study when they were accepted onto the course and given the opportunity to contact the research team to address any questions. It was made clear that their participation was voluntary and would have no impact on their status on the course. Informed consent was obtained on day 1 of the course.

Delegates from the PHEMCC were recruited across two successive years (2021 & 2022). A total of 29 delegates gave informed consent; however, full data are available for *N* = 27 following withdrawal of 2 delegates from the course in 2022. The sample comprised 9 females and 18 males (M_age_ = 36.22, SD = 6.91) of which 13 were doctors (M_age_ = 36, SD = 5; female = 4, male = 9) and 14 were paramedics (M_age_ = 37, SD = 8.3; female = 5, male = 9). Delegates had been in their current emergency medicine role for 7 years (doctors M = 8.62, SD = 3.52; paramedics M = 5.57, SD = 7.57).

### Pre-hospital emergency medicine crew course

The PHEMCC is a 2-week training course run by the Great North Air Ambulance Service (GNAAS). The course provides high-fidelity, scenario-based training for medics to develop skills and knowledge in complex, high-stress pre-hospital emergency environments and comprises life-saving medical care alongside leadership, communication strategy, stress management, and decision making.

The PHEMCC runs consecutively for 2 weeks with different activities each day, apart from Days 6 and 7 (weekend). The activities include immersive experiences and high-fidelity simulations in a range of environments with contributions from other emergency services (e.g., police, fire service, mountain rescue), and actors. Each day is accompanied by an anticipated load in terms of the learning, cognitive, and physical requirements of the activities. While week 1 largely comprises the learning and acquisition of skills, the second week involves the application of those skills in complex scenarios and as such, the anticipated loads vary across the days. Except for Day 10, where training commenced around midday and ran into the late evening, daily activities ran from approximately 08:30 h to 17:00. Daily activities and anticipated loads are summarised in Table 1.

**Table 1:** PHEMCC Daily activities and proposed learning, cognitive, and physical load

Day	Activities	Anticipated Load		
		Learning	Cognitive	Physical
Day 1	Human Factors & Pre-Hospital Emergency Assessments	H	H	L
Day 2	Surgical Skills	H	H	L
Day 3	Obstetrics, Paediatrics, Ultrasound	M-H	L	M
Day 4	Patient Transfer	M-H	M-H	L
Day 5	Water Rescue, Extreme Environments	M	M-H	H
Day 6	Weekend	-	-	-
Day 7	Weekend	-	-	-
Day 8	Road Traffic Collisions and Extrication	M	M-H	M
Day 9	Confined Spaces, Working at Height, Burns and Fire	M	H	H
Day 10	Road Traffic Collision (day-night simulation)	H	H	H
Day 11	Ballistics & Major Incidents (mass shooting simulation)	M	H	M-H
Day 12	Final Assessments	M	H	H

L = Low, M = Medium, H = High (proposed loads)

## Materials

### Psychological responding

State anxiety was measured using a version the short-form State–Trait Anxiety Inventory (SF-SA) [23]. The scale is comprised of three negative items (tense, upset, worried) and three reverse-scored positive items (calm, relaxed, content). Responses are made on a 4-point Likert scale (not at all, somewhat, moderately, very much), and all item responses are summed to give a total score, with higher scores indicating greater levels of state anxiety.

The Competitive State Anxiety Inventory (CSAI-2) [24] was used to record self-reported levels of cognitive and somatic anxiety, and self-confidence. The CSAI-2 comprises 27 items rated on a 4-point Likert scale with higher summed scores indicating higher levels of each state. It is a well-established tool for the assessment of competition anxiety across a wide variety of sporting contexts and has been previously used, alongside the measurement of cortisol, to differentiate training and competition in sport [25]. Although developed for sporting contexts, the items are relevant to stressful and challenging situations such as those experienced in emergency medicine. Only one item in the scale specifically refers to ‘competition’; in this instance competition was substituted for ‘activities’.

The Daily Anticipated Versus Experienced Demands Scale was developed for this study to assess thoughts about the forthcoming day shortly after waking, and reflection on the day’s events before sleep. The scale comprises 4 items assessing stress, worry, control, and confidence in coping, and the tense is altered to reflect thoughts before and after the day’s events. Prior to use in the current study, the items were piloted for feasibility in a sample of 7 participants taking part in the PHEMCC. The items successfully differentiated training days from non-training (weekend days), and levels of stress and worry were greater on days when perceived control and confidence in coping were lower.

### Biological responding

#### Smartwatches

Commercially available smartwatches (Garmin Vivosport or Vivosmart 5) were provided to participants. In terms of requirements for this project, the devices differed only in the presence of GPS mode on the Vivosport; however, this was not required and deactivated. The devices record a range of fitness-related indices which can be utilised directly or used to derive additional measures. Heart rate (HR) was recorded via the optical heart rate sensor on the back of the device. We also recorded stress using the Garmin Stress Index™, a proprietary index that uses HR and heart rate variability (HRV) data. Using Firstbeat analytics, the Garmin Stress Index™ uses a combination of HR and HRV data, whilst controlling for variation caused by movement. The index is scored from 0 to 100 where scores less than 25 indicate parasympathetic dominance, and scores greater than 25 indicate sympathetic dominance. Higher scores therefore indicate increasing activation of the sympathetic nervous system and therefore provide a measure of stress that is not influenced by physical movement. Devices were worn for the duration of the study and only removed for battery recharging as necessary; this process took between 20 and 30 min and was conducted during start of day briefings.

#### Salivary cortisol

Participants provided four saliva samples each day at awakening, awakening + 30 min, awakening + 45 min, and before sleep using Salivettes (Sarstedt, Germany). Samples were refrigerated by participants until collection by the researcher and were frozen (− 80◦C) until time of assay. Samples were assayed in duplicate, using the enzyme-linked immunosorbent assay method (Salimetrics Europe, Cambridge UK) at the ARU Biomarker Laboratory, Cambridge (all intra and inter assay coefficients < 10%). Samples were obtained, stored, and transported in line with the Human Tissue Act (2004). To maximise integrity of samples and adherence to protocol, saliva collection followed expert guidelines [26]. Participants were given a face-to-face demonstration of saliva provision, their first samples were supervised, and any questions addressed before they provided further samples unsupervised. Furthermore, self-reported waking times were verified using smartwatches.

### Procedure

This study was granted ethical approval by the University Ethics College. Following informed consent, on day 1, participants were provided with a smartwatch and asked to wear the device continuously for the duration of the training course. The researcher gave a demonstration of the saliva collection technique, followed by the provision of three supervised saliva sample during the training course briefing (these samples are not included in analyses as they do not align with any aspect of the proposed diurnal assessment). To minimise any impact on the training experience, with the exception of continuous measures obtained through smartwatches, all measurements were completed outside of the scheduled training activities. On each day, including the non-training (middle weekend) days, participants followed the same collection protocol. Every morning, immediately following waking, participants provided a saliva sample, followed by two more samples taken 30 min and 45 min following waking and noted their waking time and the times that samples were provided. During this sampling period participants also completed a morning questionnaire comprising CSAI-2, SF-SA, and the anticipation of forthcoming demands questions. Before sleeping on each day, participants completed the CSAI-2, SF-SA, and the experience of the day’s demands questions. Participants then provide a final saliva sample before sleep and recorded the sample time. Every day, the researcher collected all study materials from the previous day, distributed the following day’s materials, and carried out a data-synch for smartwatches. At the end of the training course, participants were given a study debrief.

### Statistical analysis

Smartwatch data were processed by Fitrockr Health Solutions (Berlin, Germany). Data collected on smartwatches were transferred at regular intervals via a Bluetooth connection to the Fitrockr Hub App on a tablet (Samsung Galaxy). Collated data were sent daily from the hub to Fitrockr and processed data for all smartwatch variables were made available via the Fitrockr Web App. To allow for consistency in analyses across all variables, continuous data (heart rate and HRV-derived stress) were aggregated to produce single values for cross-day comparisons (day 1: 09:00 h–00:00 h; Day 2 – Day 11: 00:01 h–00.00 h; Day 12: 00:01–1500 h). Salivary samples were used to derive the following indices of cortisol secretion: levels at wake, CAR magnitude (individual peak response: +30 / +45 minus awakening), and levels before sleep. Self-reported waking times corresponded with activity recorded through smartwatches. Linear mixed effects models were used to analyse all outcomes. These were fitted using the lme4R-package [27]. All models used a Gaussian likelihood with sum-coded (-1, 1) fixed effects. For psychological variables this included fixed effects of day, time (AM vs. PM), and their interaction. For physiological variables, this included a fixed effect of day only. All models contained random intercepts by participants. As there was one measurement per cell for participants this constituted the maximal random effects structure. Mixed effects models were chosen over traditional ANOVA methods as they can make accurate estimates with missing data without the need for case-wise deletion or multiple imputation. Main effects and interactions were evaluated after model fitting using ANOVA with type-III sums of squares using the Satterthwaite method for calculating degrees of freedom. After model fitting, estimated marginal means were calculated using the emmeans R-package [28]. Figures are provided of estimated marginal means where dots indicate means, ribbons indicate 95% CI, and significant differences between conditions are indicated where arrows do not overlap (*p*-values adjusted using Tukey contrasts, i.e., from the Studentised range distribution). All analyses are available at https://osf.io/r4cep/.

## Results

### Psychological responding

**Fig. 1:**
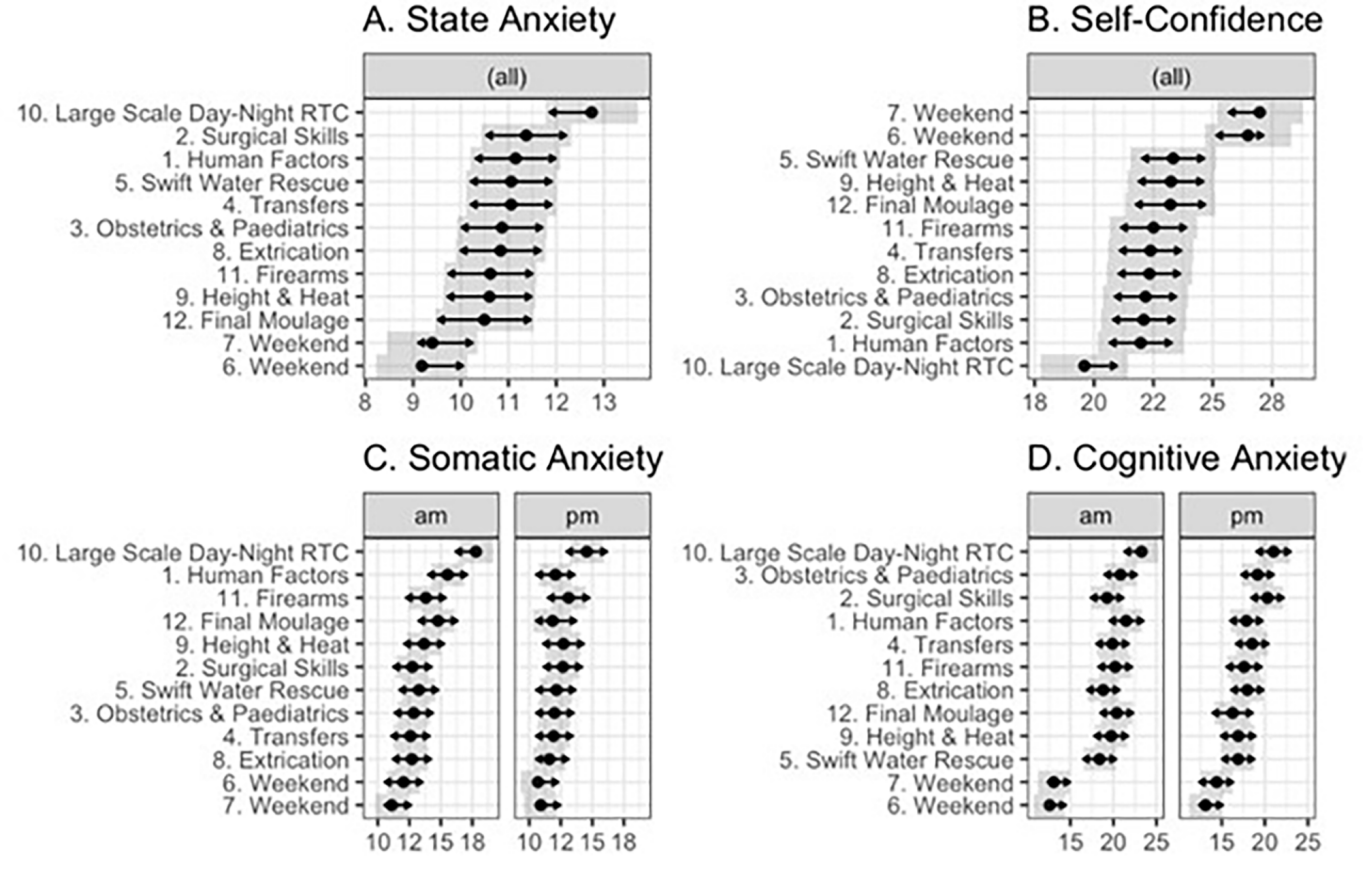
Estimated marginal means for (**A**) state anxiety, (**B**) self-confidence, (**C**) somatic anxiety, and (**D**) cognitive anxiety

There were significant main effects of day and time for State Anxiety (Day: *F*(11, 564.39) = 5.97, *p* < 0.001; Time: *F*(1,564.38) = 18.93, *p* < 0.001), Cognitive Anxiety (Day: *F*(11,565.32) = 24.09, *p* < 0.001); Time: *F*(1,565.27) = 21.27, *p* < 0.001), Somatic Anxiety (Day: *F*(11, 565.70) = 9.38, *p* < 0.001; Time *F*(1,565.61) = 30.96, *p* < 0.001), and Self-confidence (Day: *F*(11,565.14) = 12.15, *p* < 0.001; Time: *F*(1, 565.08) = 13.60, *p* < 0.001). Additionally, there were significant Day x Time interactions for Cognitive Anxiety, *F*(11,565.19) = 2.73, *p* = 0.002, and Somatic Anxiety, *F*(11,565.46) = 1.82, *p* = 0.048. Estimated marginal means for State Anxiety, Cognitive and Somatic Anxiety, and Self-confidence are presented in Fig. 1.

**Fig. 2:**
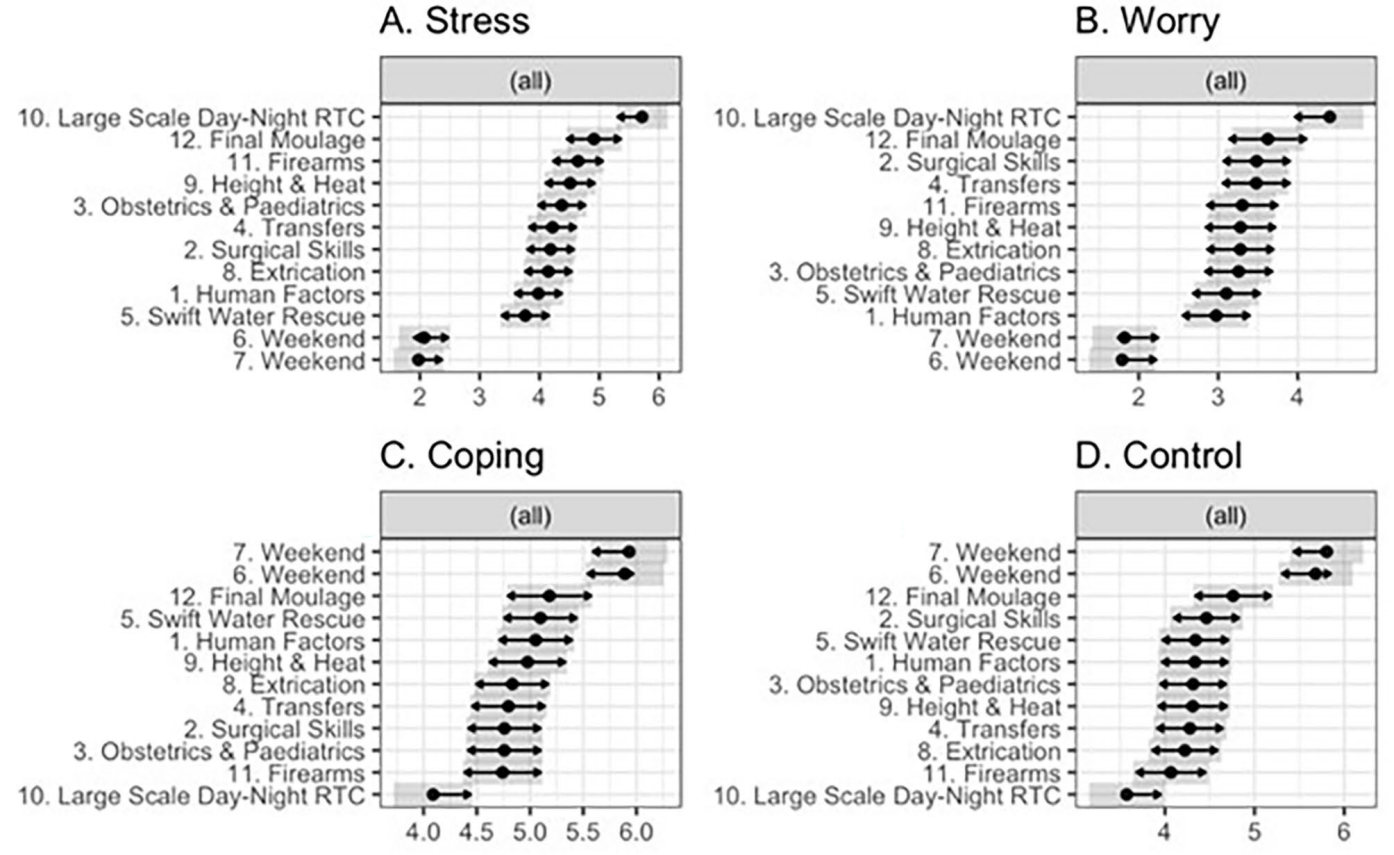
Estimated marginal means for (**A**) stress, (**B**) worry, (**C**) coping, and (**D**) control

For measures of Anticipated and Experienced Demand there were significant main effects of day and time for Stress (Day: *F*(11,563.57) = 38.08, *p* < 0.001; Time: *F*(1,563.48) = 12.22, *p* < 0.001), Worry (Day: *F*(11,563.14) = 15.17, *p* < 0.001; Time: *F*(1, 563.03) = 60.26, *p* < 0.001), Coping (Day: *F*(11, 562.43) = 11.38, *p* < 0.001; Time *F*(1,562.33) = 7.36, *p* < 0.007), and Control (Day: *F*(11,562.50) = 15.91, *p* < 0.001; Time *F*(1,562.41) = 10.73, *p* = 0.001). Estimated marginal means for measures of Anticipated and Experienced Demands are presented in Fig. 2.

### Biological responding

**Fig. 3:**
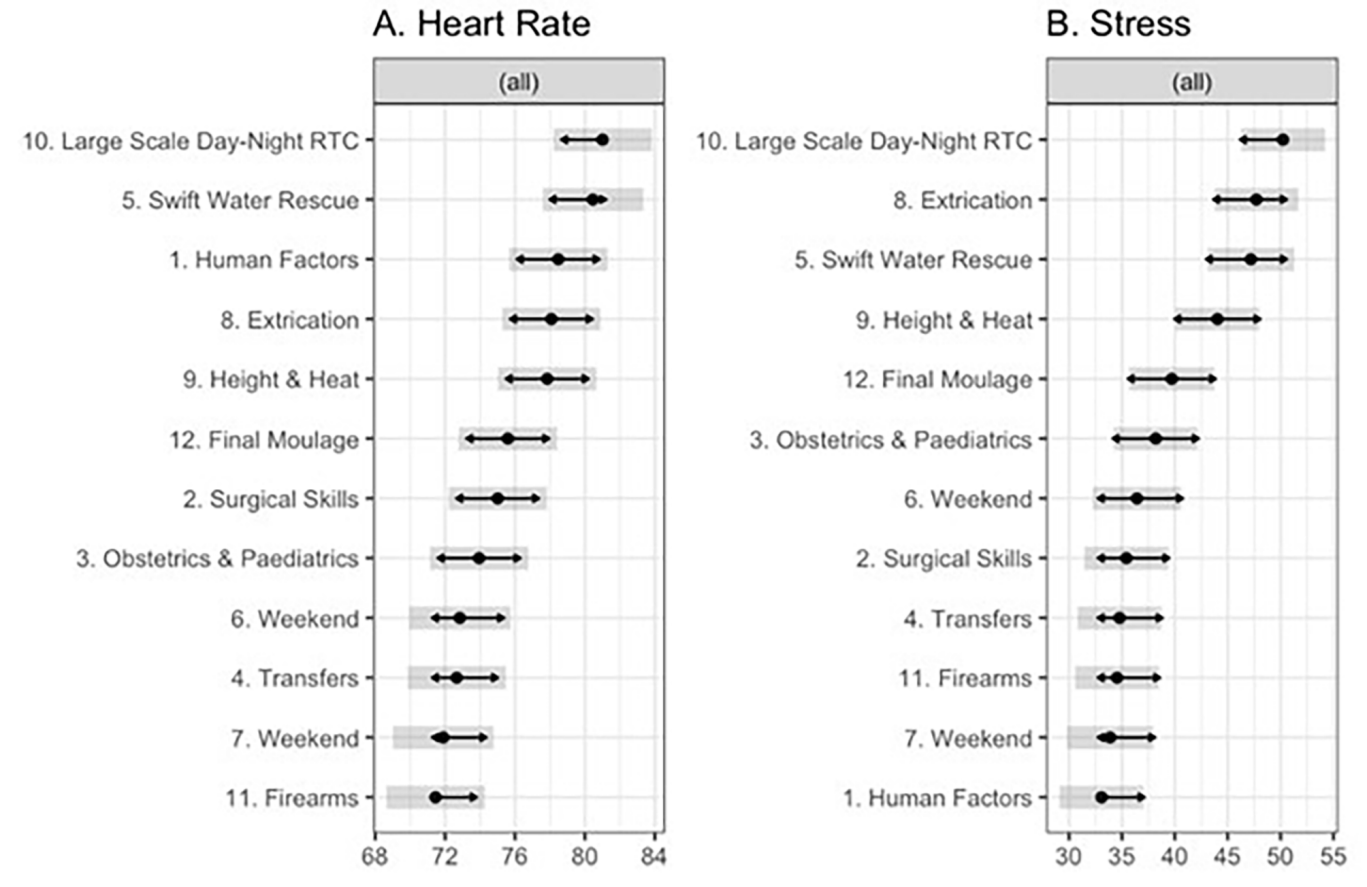
Estimated marginal means for (**A**) heart rate, and (**B**) HRV-derived stress

There were significant differences across days for aggregated measures of Heart Rate *F*(11, 256.91 = 10.50, *p* < 0.001, and HRV-derived Stress *F*(11,255.59 = 12.00, *p* < 0.001). Estimated marginal means for aggregated Heart Rate and HRV-derived Stress across days are presented in Fig. 3.

**Fig. 4:**
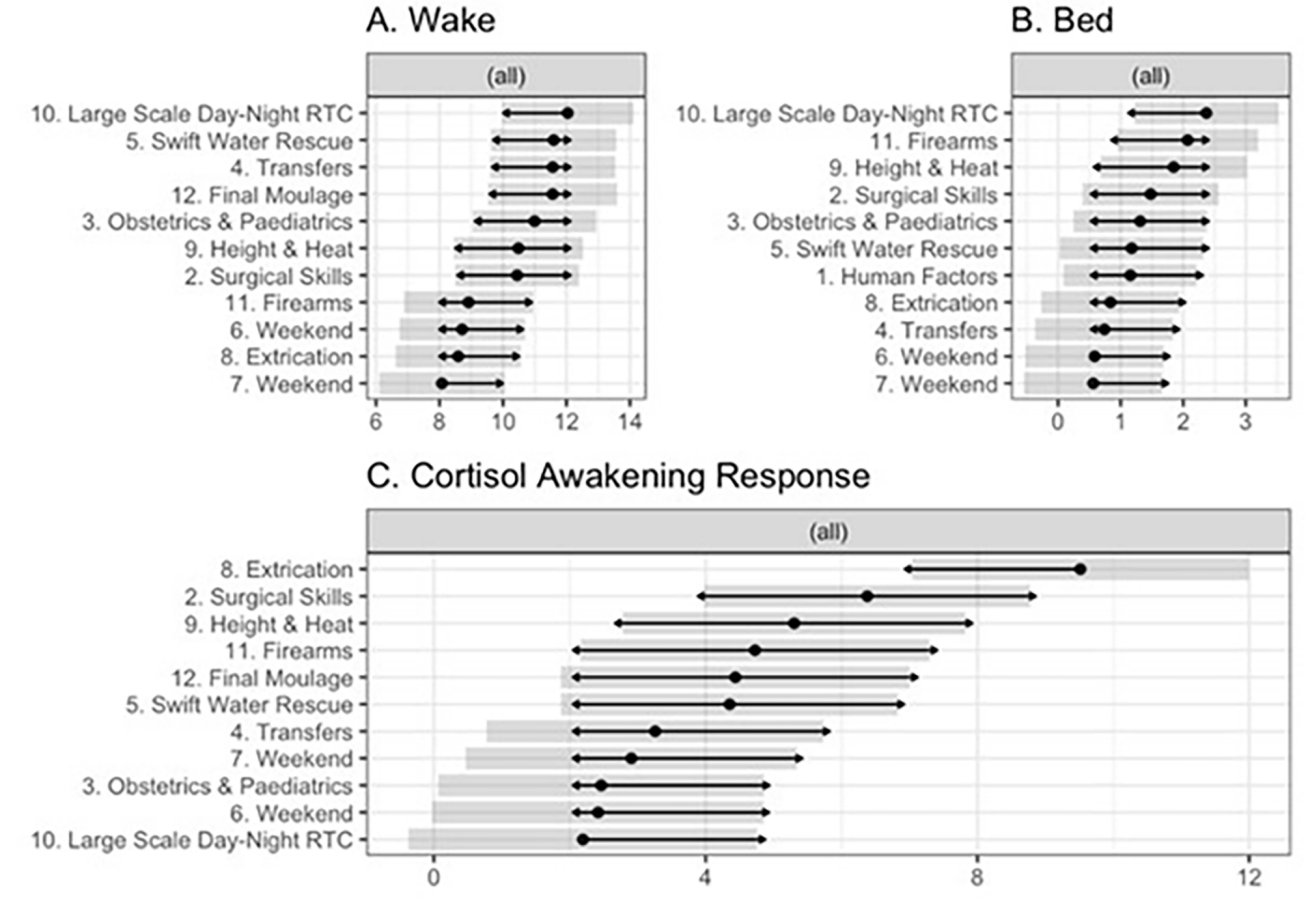
Estimated marginal means for cortisol at (**A**) wake, (**B**) bed / sleep, and (**C**) during the Cortisol Awakening Response

For cortisol indices, there were significant differences across days for aggregated measures of Waking Cortisol, F(10, 252.01) = 2.90, *p* = 0.002, and for the CAR, F(10, 248.58) = 3.8, *p* < 0.001, but not for levels before sleep, F(10, 245.96) = 1.30, *p* = 0.231. Estimated marginal means cortisol indices are presented in Fig. 4.

## Discussion

This study assessed the impact of a 2-week high-fidelity pre-hospital emergency medicine training course on psychobiological responding. Specifically, days of the training course, characterised by different learning, cognitive, and physical workloads, were compared in relation to markers of psychological and biological functioning. Analyses revealed significant differences in all variables across days, and consistent patterns with regards to the impact of different days upon psychobiological responding. The most consistent observation was a difference between training and non-training (weekend) days, where for all variables, reported levels at the weekend were statistically distinguishable from all training days. Specifically, the lowest levels of state, somatic, and cognitive anxiety, stress, and worry were reported at weekends. In contrast, compared to training days, the highest levels of self-confidence, perceived coping, and control, were reported at the weekend. Similar patterns were observed for biological measures where weekend days were among the lowest for heart rate and for levels of cortisol at Wake and during the CAR period.

While the identification of weekend, non-training days, does not speak directly to the aim of assessing the impact of high-fidelity training on psychobiological functioning, the consistent pattern of lower distress and biological responding, and higher perceived resources on non-training days, demonstrates the intended differences in load between days with training and no training. Furthermore, reduced responding at the weekend clearly demonstrates the capacity for recovery and provides further evidence of the adaptive nature of stress responding. That is, in preparation for, and during demanding, threatening, or stressful events, energy resources are mobilised via activation of the SAM and HPA axis. In contrast, in situations where perceived demand, threat, and stress are lower, the same level of resources are not required and responding can therefore be reduced. This pattern is evident during this course where the training days elicit higher levels of stress and demand and are accompanied by concomitant levels of biological responding compared with the weekend.

Although training days could be clearly differentiated from weekend days for every variable, there was less distinction across each of the training days which were characterised by similar patterns of distress and demand, and perceived coping and control. There were also similar patterns across training days for heart rate and HRV-derived stress, although there was a tendency towards greater levels of heart rate and HRV-derived stress in the second week of training. That is, for heart rate and HRV-derived stress, 4 of the top 6 highest values were recorded on days in the second week of training. The main exception was for Day 5 (Swift Water Rescue). This pattern of responding corresponds with the proposed cognitive and physical loads, where Day 5, and each day of the second training week, are categorised as medium to high load. The second week of the course involves the application of the skills developed in week 1 in complex scenarios. One day specifically however, emerged as distinct from all other training days. Day 10 (Road Traffic Collision: Day-Night) was characterised by the highest levels of state, somatic, and cognitive anxiety, stress, and worry, the lowest levels of self-confidence, coping, and control, and the highest levels of heart rate, HRV-derived stress, and cortisol levels at waking and before sleep. This day is split into two clinical scenarios which are designed to expose candidates to the demands of major incidents and mimic situations where demands exceed perceived resources. The day begins at midday rather than 9am to mimic a change in shift pattern and to facilitate a scenario that runs from dusk through to darkness. Both scenarios comprise several road traffic collisions alongside other ensuing emergencies, and involve complex decision making, multiple patients, and cross-working with additional emergency services. By this stage of the course candidates are expected to be fully immersed in the experience and care is taken to ensure that these scenarios feel as real as possible. Specific factors facilitating this realism include the use of actors, other emergency services, lighting, and simulated blood. Having a break between the two scenarios represents a gap between callouts such as would be experienced in real emergency care, and starting the second of the two scenarios at dusk adds an extra layer of realism and cognitive load. As such, this day is truly high-fidelity and is characterised by the highest learning, cognitive, and physical loads across the entire course. It is perhaps, therefore, not surprising that this day also elicits the greatest levels of psychobiological responding and the lowest levels of perceived coping and control.

The up- and down-regulation of biological responding to match perceived demand is adaptive [16], and demonstrates that the proposed learning, cognitive, and physical loads of each of the training days function as intended. These findings can be seen within the framework of the Job Demands-Resource theory [29] where increased stress arises from an imbalance between perceived work demands and job-related resources. This imbalance has been observed across a range of occupations where physical, social, or organisational aspects of a role require sustained physical and / or mental effort, and these requirements are not met with appropriate levels of resource that could buffer these demands. This is common of the lived experience of those that provide critical emergency care, where crisis situations are characterised by high levels of physical and cognitive demand, with low levels of control and perceived ability to cope [15]. The provision of emergency care is stressful, and this demonstrates the importance of high-fidelity training to also comprise the emotional elements associated with real emergency situations [18].

In line with the study aim, we have demonstrated that training days with different learning, cognitive and physical loads, are characterised by differences in psychobiological responding. Specifically, those days with the highest proposed workloads are met with the highest level of psychobiological responding. However, to what extent do these findings represent experiences in real life emergency care? High fidelity training typically comprises environments that are realistic in terms of their physical (look, feel, sight and sound) and conceptual (level of realism) attributes, while less attention is paid to emotional attributes [30]. Indeed, emotional engagement is typically greater in simulations with higher levels of realism [31]. This study demonstrates the association between emotional responses, as evidenced by stress and worry, commensurate with workloads based on high-fidelity activities, but moreover, demonstrates the subsequent impact on biological responding as evidenced by measures of heart rate, HRV-derived stress, and cortisol indices. This course comprises the key ingredients for high fidelity simulation and the psychobiological responding is therefore analogous to that which would be experienced in real emergency care. Given the high-fidelity nature of the activities and timing, Day 10 represents a typical, albeit exceptionally challenging, day that is in line with the daily requirements of front-line emergency medicine. That this day is characterised by the highest levels of psychobiological responding, whilst adaptive in the short term, may be cause for concern in the longer-term, and emphasises the importance of the opportunity for recovery to avoid the negative consequences of repeated and sustained physiological activation on health and wellbeing through the manifestation of burnout. Burnout has a significant deleterious effect on health and wellbeing through sustained activation of the nervous and endocrine systems. This activation leads to increased allostatic load and widespread dysregulation of immune, digestive, reproductive, metabolic and cardiovascular systems, alongside changes in brain structure and function [32]. Burnout most often results from a prolonged, ongoing imbalance between work demands and job-related resources, whereby work demands significantly exceed job-related resources and this is a significant problem within medical professions [33]. This is evidenced in the sector where recent reports demonstrate that over 50% of those that provide emergency medical care are experiencing moderate to high levels of burnout [34].

This study should be evaluated in light of some limitations. Although we present evidence for the high-fidelity of the training course, the overall structure, with two blocks of five training days separated by a weekend, may be less representative of the on-the-job experiences of those that deliver emergency care. For these individuals, long hours, shift work including nightshifts, and atypical working weeks are commonplace [35], and the frequency and sustained physiological activation within these work patterns will increase vulnerability to negative consequences for health and wellbeing. Within the context of this training course, the lower levels of responding observed at the weekend represent adaptive responding and demonstrate that these participants, when afforded the opportunity, experience a lowering of biological responding that matches a reduction in their perceived distress and demand. Given the significant levels of burnout in this sector, this seems less representative of their lived experience, however, the extent to which there are opportunities for adequate recovery warrants further investigation.

In support of the notion that greater CARs are associated with increased demand [13], it would be reasonable to expect the greatest CAR on Day 10. Indeed, all other markers indicate this to be the most demanding day with the highest levels of anticipated and experienced demands, and concomitantly higher levels of HR and HRV-derived stress, and cortisol before sleep. However, the CAR was not of greater magnitude on this day. Later waking is associated with smaller CARs irrespective of demands [6], and this is likely reflected in this study where on Day 10 there was a planned later start which led to later waking (over 2 h later on Day 10 than any other training day). It is therefore likely that waking time has prevented full exploration of the greatest levels of anticipated demand being associated with the greatest CAR in this study. The greatest CAR magnitude was, however, observed on Day 8. This day was characterised by similar levels of anxiety, stress, worry, control and coping to other training days; however, Day 8 is the first day of the second week of training which participants anticipate being increasingly challenging. This anticipation of forthcoming demand may drive this increased CAR in line with the view that increased CARs provide a preparatory boost at times of anticipated demand [6]. The measurement of cortisol, particularly measurements of diurnal cortisol and the CAR can be complex, and a range of factors must be considered in its collection, analyses and interpretation. It is therefore worth noting that expert guidelines [26] were followed in this study to maximise the integrity of sampling and to guide subsequent interpretations.

Heart rate and HRV-derived stress were obtained from commercially available smartwatches. There is an increasing interest in ambulatory monitoring and therefore an increased demand for non-invasive and efficient devices for the measurement of physiological variables [36]. Smartwatches may not afford the same level of accuracy as laboratory devices for some indices; however, a recent study has demonstrated that HRV derived from Garmin smartwatches provides excellent accuracy compared with gold-standard ECG-based HRV monitors [37]. Wearable devices are the most viable option for ambulatory studies and smartwatches are particularly acceptable to users. Indeed, our use of smartwatches is a novel aspect of this study design, and the same level of data collection would not have been possible without them. Another advantage afforded by the use of smartwatches is the volume of collected data. In the current study, smartwatches were worn continuously for the assessment period and these continuous data would allow for a more fine-tuned analysis of within day variations in heart rate and HRV-derived stress, including periods before, during and after the experience of stress. These analyses would be informative; however, they are beyond the scope of this paper, the main aim of which was to characterise training days by differences in psychobiological responding. As such, aggregated data were used to allow for consistent comparison. Future work could therefore consider a more detailed assessment of real-time changes in relevant indices. Future work could also consider the identification of factors that may predispose individuals to display particular patterns of psychobiological responding that may lead to negative consequences for health and wellbeing. Again, this is beyond the scope of the current study, where the sample size, which was prohibited by the capacity of the training course and the study duration, is not sufficiently powered to address questions related to individual differences.

Few studies have simultaneously assessed markers of the autonomic nervous and endocrine systems to stress in clinical or high-fidelity scenarios. Although some studies have incorporated indices of these systems [20], the focus has been on assessment surrounding a specific event rather than longer-term assessment. This study represents the longest continuous assessment of the impact of high-fidelity training scenarios in emergency care. Moreover, with the inclusion of a range of measures, this study is the most comprehensive assessment of psychobiological functioning in emergency medicine to date. It is suggested that, in addition to the simulation of environment and equipment to simulate real world scenarios, high-fidelity simulations should create realistic environments to the extent that they also elicit the emotional responses that would typically be experienced in real emergency situations [18]. This study clearly demonstrates these emotional responses, and additionally, the corresponding biological responding which would be typical in the delivery of emergency medical care.

## Conclusions

Through assessing psychobiological responding we have identified patterns of adaptive responding and recovery as evidenced by differences in responding between training days and weekends. Moreover, we have been able to link increases in cognitive and physical workloads to concomitant increases in distress, demand, and biological responding, and reductions in perceived coping and control, as evidenced by greater responding on training days with the highest loads and greatest fidelity. Given that frequent and sustained psychobiological responding, with limited opportunity for recovery, can increase the risk of burnout, and burnout is pervasive in emergency medicine, these patterns warrant further investigation.

## Data Availability

All materials are available to view at Open Science Framework (https://osf.io/r4cep/) . The data that support the findings of this study are available from the corresponding author upon reasonable request.
